# The Effect of Sliding Speed on the Tribological Properties of Ceramic Materials

**DOI:** 10.3390/ma16237252

**Published:** 2023-11-21

**Authors:** Giovanni Paolo Alparone, David Penney, Eifion Jewell, James Sullivan, Christopher Mills

**Affiliations:** 1 Department of Materials Engineering, Swansea University, Swansea SA2 8PP, UK; 2 Tata Steel, Research and Development, Swansea Technology Centre, Swansea SA2 8PP, UK

**Keywords:** ceramics, tribology, coefficient of friction, wear resistance, pin on disc, galvanizing pot hardware

## Abstract

Ceramics are considered to be candidate materials for galvanising pot bearing materials due to their excellent corrosion resistance in many molten metals. Galvanising pot roll bearings must have excellent wear resistance, and, therefore, it is important to understand the wear behaviour of prospective bearing materials. This study investigates the friction- and wear-resistant properties of select ceramics, namely, pure hBN, BN M26, AlN-BN, Macor, 3YSZ, Al_2_O_3_ and Si_3_N_4_. The ceramics were tested at different sliding speeds using a pin-on-disc device equipped with SiC pins. The lowest coefficient of friction (COF) achieved was below 0.1, and it was measured for pure hBN at a 3.14 m/min sliding speed. However, a wear scar analysis showed that the BN grades suffered from severe wear. The highest wear rate was obtained for BN M26 at a 9.42 m/min sliding speed and was equal to 17.1 × 10^−6^ mm^3^ N^−1^ m^−1^. It was shown that the wear coefficient of the tested ceramics varied exponentially with hardness. The lowest wear was observed on the 3YSZ, Al_2_O_3_ and Si_3_N_4_ ceramics, which showed no volume loss, and, for this reason, they can be potentially used as bearing materials in continuous galvanising lines.

## 1. Introduction

Ceramics have been reported to be good candidates for many wear-resistance applications due to their high compressive strength, high hardness, high elastic modulus and excellent resistance to oxidation and creep [1,2]. Zirconia is typically used in the gears of heat engines or in the biomedical field, for example, to make femoral ball heads [1,3]. Silicon nitride balls can be found in turbo pumps, and silicon carbide is used in bulletproof-vest assembly units [1,4]. Furthermore, cermets based on TiCN are found in the cutting-tool inserts used in high-speed machining [1].

Recently, the properties of ceramic materials have attracted the attention of the galvanising industry, which is investigating their use as galvanising pot bearing materials. The pot hardware used in continuous galvanising lines comprises submerged rolls, which guide the steel in the zinc pot. The key components of the galvanising bath hardware are the journal bearings, whose lifetime is very limited and typically does not exceed five weeks. The reaction of the bearing materials with the molten zinc alloy bath is responsible for the deterioration of these components, causing quality issues and unplanned line stops. The hardware materials currently in use include Co-based alloys and WC/Co thermal spray coatings [5]. However, ceramics have the potential to outperform these materials due to their chemical inertness. Therefore, recent research has focused on testing ceramics as pot bearing materials. For example, oxides such as Al_2_O_3_ and ZrO_2_ were reported not to react with liquid zinc [6,7]. Other ceramic materials, including BN and silicates have been applied to galvanising pot rolls to enhance the corrosion resistance of the roll materials in liquid zinc [8]. In addition to this, nitrite ceramics, such as SiAlON have been tested in liquid zinc to analyse their reactivity and wear behaviour in a molten zinc alloy [9,10].

The aim of this work is to discuss the tribological properties of ceramics with the potential to replace the bearing materials used in continuous galvanising lines. The wear resistance of metals has been extensively discussed in the literature; for instance, experiments were performed to determine the influence of certain heat treatments on the wear behaviour of steel [11,12,13]. However, there is a lack of comparative studies on technical ceramics [14]. Wear testing was conducted on composite materials containing BN [15,16,17,18,19,20,21,22,23,24,25,26,27,28,29], although less information is available regarding the tribological properties of pure BN specimens. Several of these studies performed tests to analyse the impact of BN additions on the friction- and wear-resistant properties of different materials [16,17,19,21,25,26,27,28,29]. No previous work was conducted on BN-SiO_2_ composites, such as BN-grade M26, in the subject of tribology. AlN-BN composites, which are known for their good machinability, were studied, and their mechanical properties, including hardness, were discussed [30,31,32]. Little information was provided on the wear properties of AlN-BN.

Glass ceramics, such as Macor, were tested to study the cavitation of the material in an aqueous solution [14]. This type of wear was less likely to occur on the hardware submerged in the galvanising bath. Further wear tests were performed on Macor glass ceramics using polymeric bearings as their counterface [33]. Regarding Si_3_N_4_ ceramics, the effect of the BN inclusions in Si_3_N_4_-BN composites on the wear properties was investigated [28,34]. Work involving the testing of pure Si_3_N_4_ specimens was carried out in environments where lubricants, such as water, were used [35,36]. In addition to this, wear tests were performed using steel counterfaces [37,38]. However, the hardness of steel is expected to not be sufficiently high to produce wear on hard ceramics, such as Si_3_N_4_. The wear behaviour of Al_2_O_3_ and ZrO_2_ was determined using counterfaces of the same material [39,40]. The bearing components used in galvanising baths are chosen so that one material is sacrificial to the other. Therefore, if testing is performed on pot roll bearing materials, the use of a counterface with a different hardness is required.

The present work compares the tribological properties of select ceramics tested against a SiC counterface. Tribological properties are typically investigated using tribometers such as the pin-on-disc, four-ball and Timken apparatuses [28,41,42,43]. A limitation of the four-ball and Timken tribometers is that the contact stress can vary during testing. In this study, friction and wear characterisation was performed using a pin-on-disc tribometer, which offers better control of the experimental conditions compared to the other two techniques [42]. Due to the wide selection of prospective bearing materials and the lack of comparative studies available in the literature, it was necessary to test these materials at the same sliding conditions. This allowed us to compare the wear behaviour of the materials and to provide valuable preliminary data to galvanisers who wish to perform experiments, e.g., to determine statistical variations.

## 2. Materials and Methods

**Figure 1:**
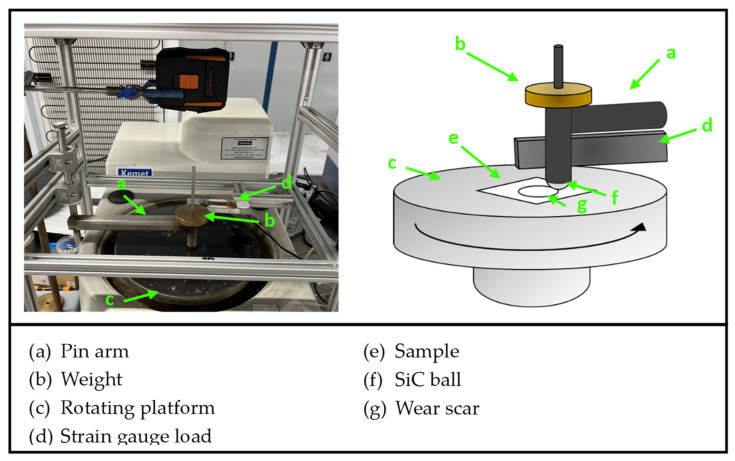
Pin-on-disc tribometer.

Friction and wear testing was performed on seven different ceramic materials procured from Precision Ceramics, Ltd. (Birmingham, UK). The name of the materials as well as their composition can be found in Table 1. The samples consisted of 80 × 80 × 5 mm plates, which were polished to a roughness of <1 µm (Ra) using a diamond slurry before testing. Silicon carbide (SiC) ball bearings with a hardness of 2200–2800 HV and 10 mm diameter were used as the pin [44]. SiC was chosen due to its superior hardness compared to that of the ceramic ‘discs’ tested, making the disc the expected sacrificial counterpart. The experiment was carried out using a bespoke pin-on-disc apparatus at Swansea University, which is illustrated in Figure 1. The setup consisted of a rig where an arm was weighted down onto the ceramic specimens with a known constant load of 2 N to press the pin against the rotating specimen, as shown in a previous work using the same pin-on-disc system [45]. Higher loads were avoided to prevent the softer ceramics from breaking during the tests. The SiC sphere was attached to the arm, as shown in the figure, and the applied force was controlled by adding weights to the ball holder. The rig was fitted with a strain gauge load cell, which measured the friction force upon contact with the pin arm. The friction force was recorded automatically by the data processing software. The pin-on-disc apparatus was positioned above the rotating platform of a polishing machine, where the test sample was fixed. Testing was performed in the upper-pin-on-bottom-disc mode without lubrication and at room temperature. The rotations of the platform were set at 50 rpm, 100 rpm and 150 rpm, which, respectively, corresponded to 3.14 m/min, 6.28 m/min and 9.42 m/min sliding speeds, i.e., the speed at which the ceramic plate slid against the SiC pin. The duration of sliding contact was 20 min, and the ball bearing was replaced before starting each test so that all the ceramic ‘discs’ were tested against a new SiC pin.

The ceramic samples were visually inspected and characterised after testing to analyse the wear scars. Images were captured using a Keyence (Osaka, Japan) VHX-7000N digital microscope. To determine the wear loss of the samples, 3D scans of the wear scars were recorded using a surface profilometer. The wear volume loss of the discs *V* was obtained according to Equation (1):

(1) V=A×L,

where ‘*A*’ is the measured cross-sectional area of the wear scar and where ‘*L*’ is the length of the stroke and is equal to the circumference of the circular trace left after the test. The worn track section ‘*A*’ was obtained by performing a line scan across the worn track. The total sliding distance ‘*S*’ was obtained by multiplying the sliding speed ‘*v*’ in m/min with the duration of the test ‘*t*’ in min (Equation (2)):

(2) S=v×t,

This value was subsequently used to calculate the wear rate *k*, as shown by Equation (3) [27]:

(3) $$k=\frac{V}{F\times S\;}[{mm}^{3}/Nm],$$

where ‘*F*’ is the normal contact load and ‘*S*’ is the sliding distance.

The hardness of all the ceramic samples examined in this work was tested using an Innovatest hardness machine, and all hardness readings were measured via Vickers Hardness (HV). Ten measurements were taken on each ceramic material, and indentation loads between 3 and 5 kgf were used. Bulk hardness was performed to minimise the dependence of microhardness of certain ceramics, such as YSZ, on the indentation load [46].

## 3. Results and Discussion

### 3.1. Hardness Testing

The average hardness values of the ceramics tested in this study are summarised in Table 2. The results show that the range of hardness values measured was very wide, and, therefore, it is possible to divide these ceramics into three groups. The first group includes pure hBN and BN-grade M26, whose hardness was very low (<20 kgf/mm^2^). The type of boron nitride used in these experiments was the hexagonal form, where the atoms were arranged in a structure that resembles that of graphite, consisting of atomically flat layers of B and N atoms bound together by van der Waals forces between the layers [47]. This structural analogy with carbon was also observed in the properties of the material, as the hexagonal BN was soft like graphite [48]. The second group of ceramics includes those with high hardness values, such as 3YSZ, Si_3_N_4_ and Al_2_O_3_. Among the ceramics tested, the hardest material was Al_2_O_3_, whose hardness was measured to be around 1680 HV. The remaining materials, Macor and AlN-BN, fall into the third group, as their hardness was intermediate, falling between the soft BN grades and hard ceramics like Al_2_O_3_.

### 3.2. Wear Testing

#### 3.2.1. Measurements of Friction Coefficient

**Figure 2:**
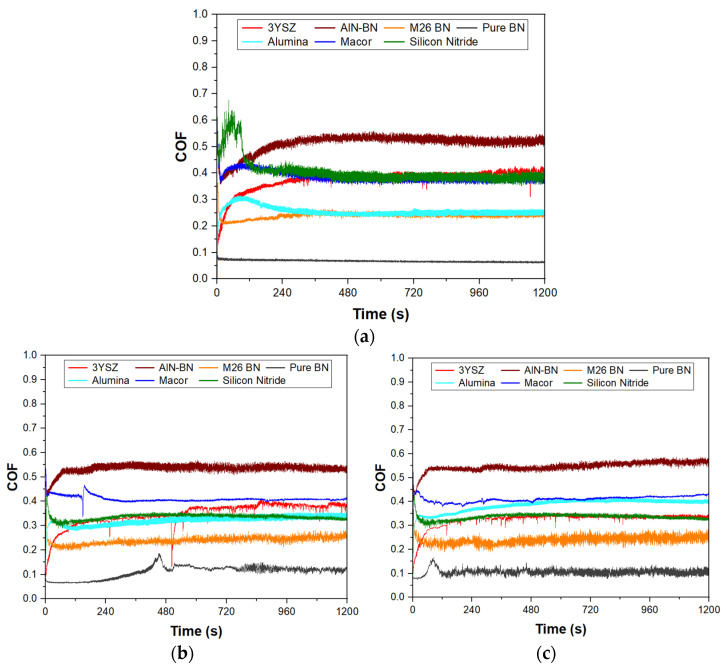
COF curves for the tests performed at (**a**) 3.14 m/min, (**b**) 6.28 m/min and (**c**) 9.42 m/min.

The coefficient of friction (COF) was plotted against the sliding time, and the friction coefficient curves obtained at 3.14 m/min, 6.28 m/min and 9.42 m/min speeds are shown in Figure 2. It is possible to notice that the COF varied with time until it reached constant values, which is a behaviour typically associated with a steady state of tribological conditions [49]. The values of the COF presented in this study were extrapolated from this region. As the ceramics reached a steady state at different sliding times, averages of the COF were taken between 960 and 1200 s (Table 3), when the COF was constant for all the ceramics.

The curves obtained at a 3.14 m/min sliding speed, Figure 2a, show that the COF of 3YSZ and AlN-BN increased slowly with time until a maximum value was reached when a steady state occurred. For the pure hBN specimen, the steady-state regime was immediately reached as soon as sliding with the SiC pin occurred. Si_3_N_4_, Al_2_O_3_, Macor and M26 BN showed a different behaviour, as the COF increased more rapidly and the maximum peak was reached at shorter sliding times. This was followed by a sudden drop in the COF values, which subsequently stabilised as steady-state conditions were approached. The COF of Macor increased again after the first drop to reach a second peak before decreasing to the steady-state values. Similar behaviours were observed when the sliding speed was increased to 6.28 m/min and 9.42 m/min, as illustrated in Figure 2b,c, except for those of pure hBN, whose COF reached a maximum peak followed by a rapid decrease.

When sliding was initiated, the increase in the COF values was due to an increased interaction between the asperities of the two materials, as the surface layer of the ‘disc’ was removed. The COF reached a peak when maximum adhesion was achieved, which occurred when the asperities in the material deformed and the wear rate was increased due to a higher presence of worn particles. If the asperity deformation processes decreased after reaching the maximum peak, the COF dropped until the interfacial steady state of tribological conditions was achieved [49]. At a 3.14 m/min sliding velocity, the ceramic which displayed the lowest friction with the SiC ball was pure hBN. The COF recorded for this material was 0.06, which is significantly lower compared to the COF values recorded for the other ceramic materials tested in this study. The hexagonal form of BN is known for its ability to provide lubrication as a result of the sliding of the hBN layers that are held by weak Van der Waals interactions [27,28,50]. Other studies have confirmed that the COF of pure hBN can assume values less than 0.1, which is a distinct feature of lubricious materials [51,52]. Moreover, Figure 2 shows that the steady-state condition was reached immediately, which may be the result of the formation of a tribolayer between the two contacting surfaces as soon as sliding with the SiC pin was initiated. BN-grade M26 also showed a low COF, although it stabilised to a constant value of 0.24 at a 3.14 m/min sliding speed. This value is higher than that recorded for pure hBN due to the presence of SiO_2_ in the material, which is believed to increase the friction with the SiC pin.

Among the three ceramics containing BN, the AlN-BN composite is the material with the largest COF, which was recorded to be 0.52 at a 3.14 m/min sliding speed. In addition to this, the COF was higher than those recorded for all the other ceramics, and it is theorised that the large difference in hardness with the SiC counterpart led to higher friction. Although several studies confirm that the tribological properties of materials can be improved by BN additions to their structure [25,26,27,28,29], in this case, BN did not provide sufficient lubrication to the system in question. This behaviour could be related to the composition of the ceramic, as the formation of a protective tribolayer in a material is reported to be linked to the amount of BN present [29].

Si_3_N_4_, Macor and 3YSZ showed a similar COF at 3.14 m/min and when a steady state was reached. The COF was measured to be around 0.4 for 3YSZ and 0.38 for Macor and Si_3_N_4_. Regarding Al_2_O_3_, although the COF recoded was higher than that of pure hBN, it matched the values obtained for BN-grade M26. Therefore, low friction was observed in the Al_2_O_3_ ceramic at the lowest sliding speed, and this behaviour was observed without benefitting from the lubrication effect provided by hBN.

**Figure 3:**
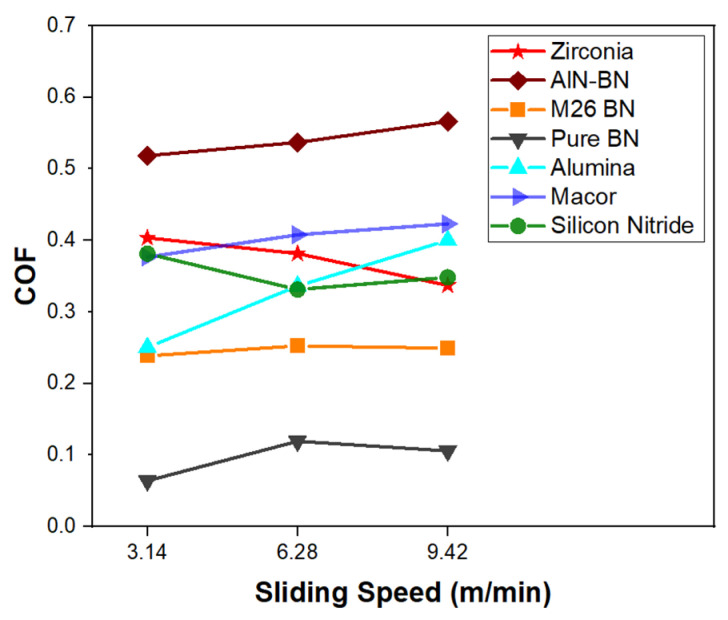
COF as a function of sliding speed.

As the sliding speed was increased to 6.28 m/min and 9.42 m/min, changes in the COF of the ceramics were observed. Figure 3 shows the COF as a function of the sliding velocity for all the materials tested. The COF of pure hBN increased from 0.06 to values slightly higher than 0.1. However, the COF value recorded was still low, considering that the specimen was sliding at a rate three times higher than the first test. The COF of BN M26 showed minimal variations with velocity, and, therefore, the lubrication provided by BN seemed to not be affected by the higher sliding speeds. On the other hand, an increased friction value was observed for AlN-BN, which remained the material with the highest COF among the ceramics tested. The same trend was followed by Macor and Al_2_O_3_, with Al_2_O_3_ being the material that most severely suffered from the higher sliding velocities. The COF no longer matched the values recorded for BN M26 and was found to increase to 0.34 and 0.4 at 6.28 m/min and 9.42 m/min, respectively. The opposite trend was observed for Si_3_N_4_ and 3YSZ, as the COF decreased from the values recorded at the lowest sliding speed. Due to its low thermal conductivity, 3YSZ was expected to suffer the high velocities worse compared to the ceramics with higher thermal conductivity (e.g., Al_2_O_3_), which can better dissipate the higher heat from the contact caused by the higher speed [1,53]. However, the results collected in this work show that the COF of 3YSZ decreased from 0.4 at 3.14 m/min to 0.34 at 9.42 m/min, indicating that the friction of 3YSZ with the SiC counterface was reduced at high speeds.

#### 3.2.2. Inspection of the Tested Specimens

**Figure 4:**
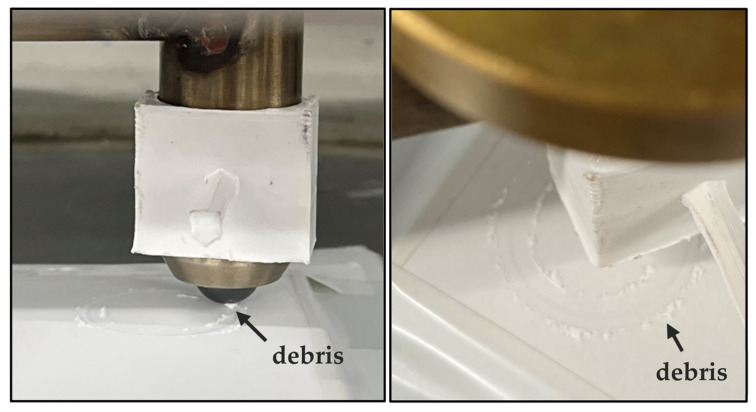
Macroscopic examination of the tested specimens.

Debris formation was observed when the specimens were visually inspected during testing. Debris accumulated on the SiC pin as well as on each side of the wear track, as shown in Figure 4. The photographs were taken on a pure hBN specimen during testing, and more debris accumulated as the sliding time was increased. Debris formation was observed on hBN, BN M26, AlN-BN and Macor. No debris was observed on a macroscopic level when 3YSZ, Al_2_O_3_ and Si_3_N_4_ were tested.

#### 3.2.3. Wear Scar Analysis

**Figure 5:**
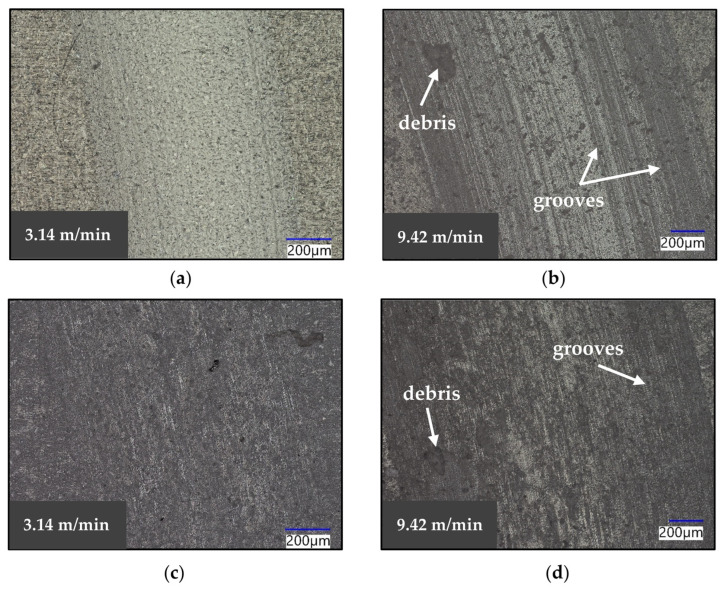
Optical images of the wear scars of (**a**) pure hBN at 3.14 m/min, (**b**) pure hBN at 9.42 m/min, (**c**) BN M26 at 3.14 m/min and (**d**) BN M26 at 9.42 m/min.

Images of the wear scars left on the ceramics at 3.14 m/min and at 9.42 m/min were compared. Figure 5 shows the surfaces of pure hBN and BN-grade M26 after testing. On the pure hBN sample, it is possible to notice the appearance of multiple parallel grooves when the sliding velocity was increased to 9.42 m/min (Figure 5b). The grooves were the result of the cutting of the softer hBN specimen by the hard asperities present in the SiC pin. Multiple grooves formed due to the presence of multiple asperities in contact at the interface. The cut material was removed as wear debris, which could also be found inside the wear track, and it was mainly present on the sample tested at a high speed. For the BN M26 specimen, grooving started to occur at a 3.14 m/min sliding speed, and a large amount of debris could be found inside the wear track, suggesting that the grooves provided space for particle entrapment (Figure 5c,d). The width of the scar on the two BN grades was found to increase when higher velocities were used. The width of the scar on the pure hBN ceramic increased from approximately 885 µm to 1660 µm as the speed was varied from 3.14 m/min to 9.42 m/min. Regarding BN M26, the width increased from approximately 1010 µm to 1940 µm, suggesting that the BN grades suffered from severe wear, particularly at a high speed.

**Figure 6:**
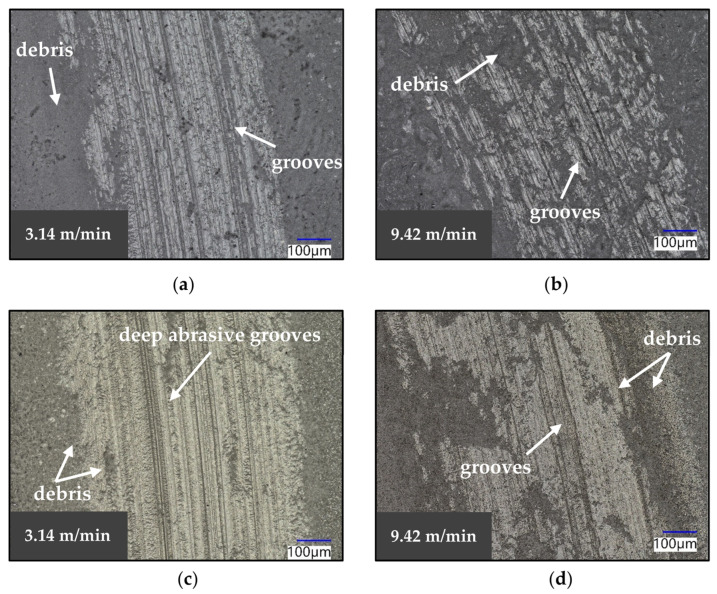
Optical images of the wear scars of (**a**) Macor at 3.14 m/min, (**b**) Macor at 9.42 m/min, (**c**) AlN-BN at 3.14 m/min and (**d**) AlN-BN at 9.42 m/min.

Figure 6a,b respectively show the surface of Macor and the AlN-BN composite. Both of the two materials showed evidence of ploughing, as deep grooves were observed at 3.14 m/min and at 9.42 m/min sliding speeds. Large amounts of debris were found on the examined surface, which deposited on the sides as well as on the inside of the wear scar. Furthermore, debris entrapment was more pronounced on the specimens tested at a high sliding speed. On the AlN-BN ceramic, the width of the scar increased slightly from around 662 µm at 3.14 m/min to 876 µm at 9.42 m/min. A similar behaviour was observed on the Macor specimen, as the width increased from around 530 µm to 671 µm when the sliding speed was increased to 9.42 m/min.

**Figure 7:**
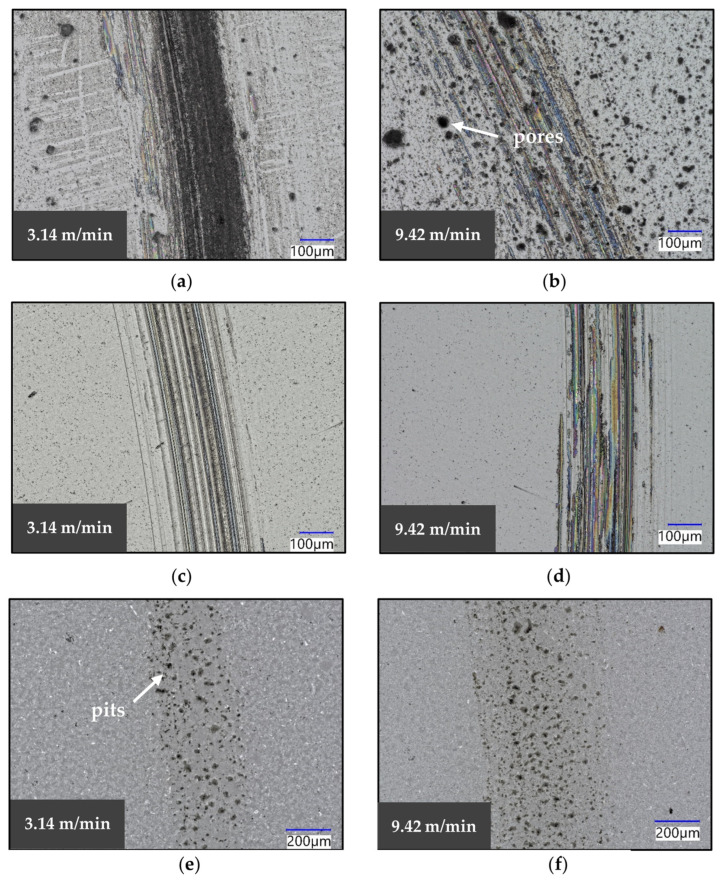
Images of the wear scars of (**a**) 3YSZ at 3.14 m/min, (**b**) 3YSZ at 9.42 m/min, (**c**) Si_3_N_4_ at 3.14 m/min, (**d**) Si_3_N_4_ at 9.42 m/min, (**e**) Al_2_O_3_ at 3.14 m/min and (**f**) Al_2_O_3_ at 9.42 m/min.

**Figure 8:**
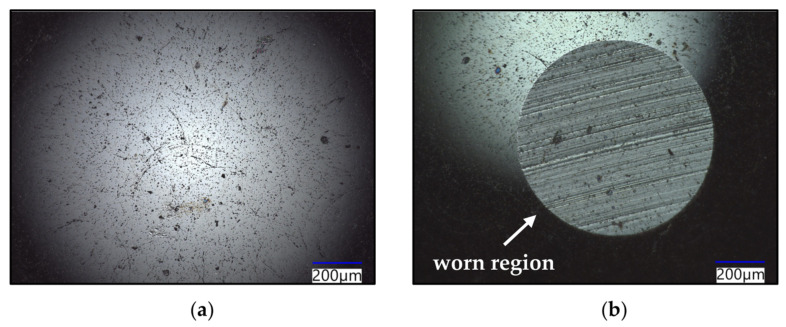
Images of the SiC ball before testing (**a**) and after contact with Al_2_O_3_ (**b**). The ball was worn during sliding, forming a ‘flat-on-flat’ contact.

The wear tracks left on the 3YSZ, Si_3_N_4_ and Al_2_O_3_ specimens are shown in Figure 7. Several differences can be noticed compared to the images previously discussed. The features of the wear scar do not appear as distinct, except for the presence of scratch marks on the 3YSZ (Figure 7a,b) and Si_3_N_4_ (Figure 7c,d) specimens, and small pits are on the Al_2_O_3_ sample (Figure 7e,f). The scratches are oriented with the sliding direction, and, therefore, they are believed to have been caused by the SiC ball, which was worn by the harder ceramics tested in this study, as show in Figure 8. It is postulated that the flattening of the ball resulted in the increase in the COF of Al_2_O_3_ observed at the higher sliding speeds, as reported in Section 3.2.1. Figure 7e,f show that the width of the scar increased from around 330 µm at 3.14 m/min to approximately 570 µm at 9.42 m/min, and it is believed that the increase in the surface in contact was due to the flattening of the counterface. The images captured of 3YSZ and Si_3_N_4_ showed that the scratching became less severe at high sliding speeds, suggesting a reduced interaction between the asperities of the two materials. The COF dropped when the asperity deformation processes decreased, as previously explained. Therefore, it is postulated that this reduced interaction observed at 9.42 m/min resulted in a decrease in the COF of 3YSZ and Si_3_N_4_ as the speed was increased. In addition to this, it was noticed that the surface of the as-received Si_3_N_4_ specimens contained a considerable amount of pores, and their presence could be related to the sintering process conditions used to manufacture the plates. The ceramics were procured as finished components from the supplier, and, for this reason, the origin of these features could not be identified with the information available. The absence of debris on the surface of the materials after testing suggests that the wear on these ceramics was less severe.

**Figure 9:**
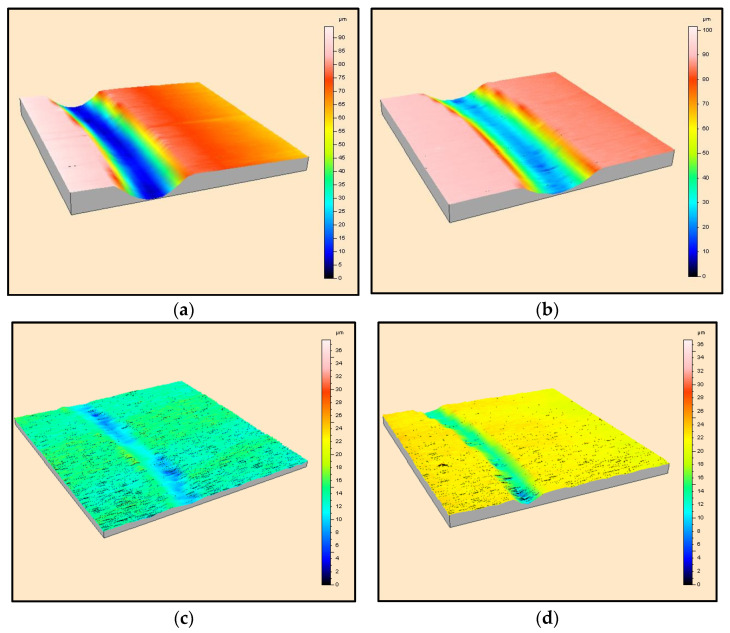
3D scans of the worn surface of (**a**) pure hBN, (**b**) BN M26, (**c**) Macor and (**d**) AlN-BN at 9.42 m/min sliding velocity.

**Figure 10:**
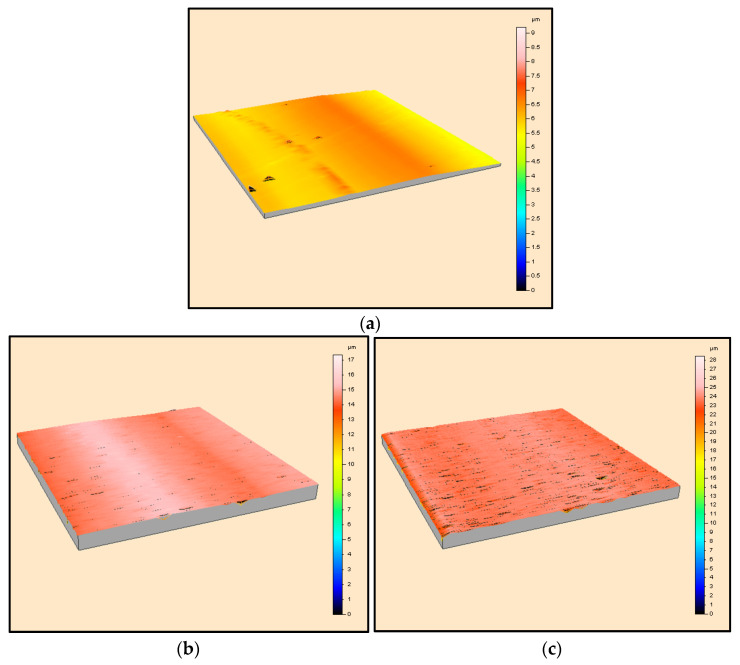
3D scans of the worn surface of (**a**) 3YSZ, (**b**) Si_3_N_4_ and (**c**) Al_2_O_3_ at 9.42 m/min sliding velocity.

To further investigate the wear scar morphology, 3D scans of the worn surface were obtained for the ceramics tested at 9.42 m/min. Figure 9 shows the scans obtained for the soft ceramics, i.e., hBN, BN M26, AlN-BN and Macor, whereas those generated for the harder ceramics, namely, Al_2_O_3_, 3YSZ and Si_3_N_4_, can be found in Figure 10. The scans confirmed that the soft BN grades suffered from severe wear, as the scar was wide and deep (Figure 9a,b). The worn surface decreased on the Macor and AlN-BN specimens, and the scar was narrower and less deep, as shown in Figure 9c,d. On the other hand, Al_2_O_3_, 3YSZ and Si_3_N_4_ did not develop a ‘channel’ upon sliding with the SiC pin (Figure 10a–c).

**Figure 11:**
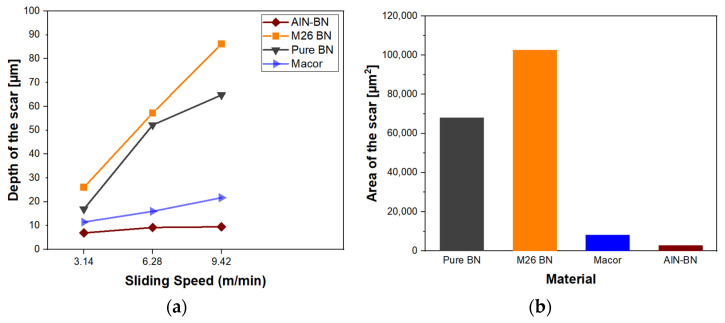
Depth of the worn track section against sliding speed (**a**). Measured cross-sectional area of the wear scar at 9.42 m/min sliding speed (**b**).

The depth of the scar was measured on the ceramics that suffered from severe volume loss after testing (Table 4), and the depths were plotted against the three different sliding velocities (Figure 11a). The measured cross-sectional areas for the materials tested at a 9.42 m/min sliding velocity are plotted in Figure 11b. The measurements confirmed that pure hBN and BN M26 were the materials with more damage, and they were also the ceramics which most suffered from the higher sliding speeds. Figure 11a shows that there was a steeper increase in the scar depth with speed for the pure hBN and BN M26 specimens compared to Macor and AlN-BN.

#### 3.2.4. Wear Resistance

**Figure 12:**
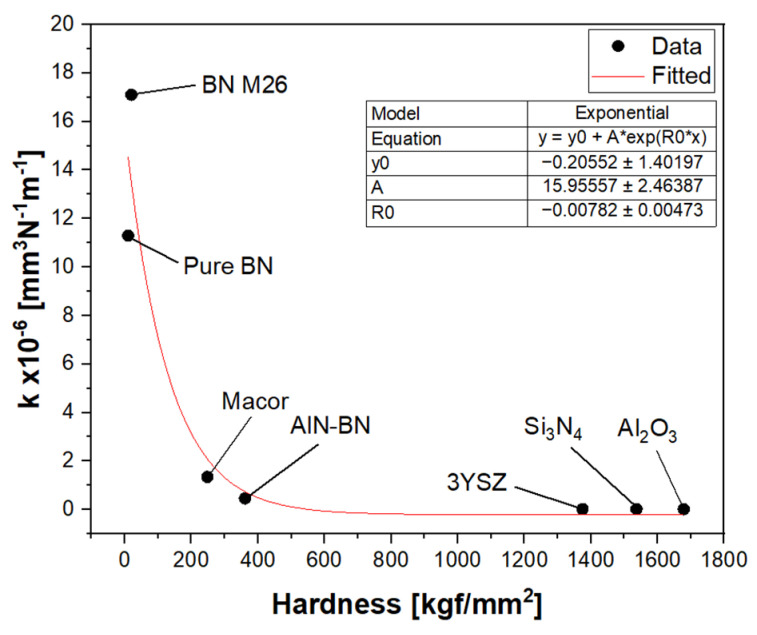
Plot of the wear coefficient ‘*k*’ against the hardness of the ceramics.

The wear of the ceramics was measured using the wear coefficient ‘*k*’, which was determined in terms of volume loss per unit force per unit distance, as shown by Equation (3) in Section 2. The higher the wear coefficient, the less the wear resistance of the material. The values of *k* for all the ceramics tested at 9.42 m/min are provided in Table 5. The wear coefficients of Al_2_O_3_, 3YSZ and Si_3_N_4_ were significantly lower compared to the softer materials, as minimal volume loss was detected under the conditions stated here, and they are considered to have superior wear resistance. The ceramics with the highest wear were the two BN grades, which were severely damaged by the harder SiC pin. The wear coefficients at a 9.42 m/min sliding speed were plotted against the hardness of the materials (Figure 12). There was a correlation between these two material properties. The softer materials, namely, pure hBN and BN M26, displayed poor wear resistance compared to the hard ceramics, Al_2_O_3_, 3YSZ and Si_3_N_4_. The hardness of AlN-BN and Macor was intermediate, falling between the low hardness of hBN and the high hardness of Al_2_O_3_, 3YSZ and Si_3_N_4_, and, therefore, they showed moderate wear. Moreover, the graph suggests that there is an exponential relationship between the wear rate and hardness; however, more experiments need to be performed to confirm this trend.

## 4. Conclusions

In this study, the tribological properties of select ceramics were investigated by performing a pin-on-disc test at different sliding velocities. The following information was concluded:

- Among the ceramics tested, hexagonal BN had the lowest COF, which reached values < 0.1 at a low sliding speed. This result confirmed that hexagonal BN is a lubricious material. Al_2_O_3_ showed a low COF with the SiC pin at a low sliding speed, whereas Si_3_N_4_ and 3YSZ showed a moderate COF at high sliding speeds.
- The wear scar of the soft ceramic materials was deeper, and the depth was observed to increase with the sliding speed. The scar was characterised by a large amount of debris, and evidence of grooving was found. However, no debris was detected in the harder ceramics, whose wear track was predominantly characterised by scratches on the surface of the material.
- The wear rate of the ceramics was found to be related to their hardness, as the soft ceramics, such as the BN grades, showed poor wear resistance. Macor and the AlN-BN composite suffered from moderate wear, whereas 3YSZ, Al_2_O_3_ and Si_3_N_4_ showed excellent wear resistance upon sliding with the SiC pin.

These preliminary results serve as a down select for further investigations where ceramics will be tested statically and dynamically in liquid zinc alloys.

## Figures and Tables

**Table 1:** Composition of ceramic plates.

Name	Composition
Pure boron nitride (hexagonal)	hBN
Boron-nitride-grade M26	40 wt.% SiO_2_, 60 wt.% BN
Macor machinable glass ceramic	46 wt.% SiO_2_, 17% MgO, 16% Al_2_O_3_, 10% K_2_O, 7% B_2_O
AlN-BN composite	AlN, BN
Yttria-stabilised zirconia (3YSZ)	3 mol.% Y_2_O_3_-ZrO_2_
Silicon nitride	Si_3_N_4_
Alumina	Al_2_O_3_

**Table 2:** Hardness values of the ceramic plates. The applied test load is reported in ‘kgf’ next to each numeric hardness value.

Name	Hardness
Pure boron nitride (hexagonal)	10 ± 1 HV/4
Boron-nitride-grade M26	20 ± 2 HV/4
Macor machinable glass ceramic	248 ± 27 HV/5
AlN-BN composite	361 ± 25 HV/4
Yttria-stabilised zirconia (3YSZ)	1375 ± 8 HV/3
Silicon nitride	1537 ± 16 HV/3
Alumina	1679 ± 10 HV/3

**Table 3:** COF of the ceramics during steady-state regime.

Material	3.14 m/min	6.28 m/min	9.42 m/min
Pure hBN	0.06 ± 0.00	0.12 ± 0.01	0.11 ± 0.01
M26 BN	0.24 ± 0.00	0.25 ± 0.01	0.25 ± 0.01
Macor	0.38 ± 0.01	0.41 ± 0.00	0.42 ± 0.00
AlN-BN	0.52 ± 0.01	0.54 ± 0.01	0.57 ± 0.01
3YSZ	0.40 ± 0.01	0.38 ± 0.01	0.34 ± 0.00
Si_3_N_4_	0.38 ± 0.01	0.33 ± 0.00	0.35 ± 0.00
Al_2_O_3_	0.25 ± 0.01	0.34 ± 0.01	0.40 ± 0.01

**Table 4:** Measurements of the wear scar depths.

Material	3.14 m/min	6.28 m/min	9.42 m/min
Pure hBN	16.9 µm	52.2 µm	64.8 µm
M26 BN	26.0 µm	57.2 µm	86.2 µm
Macor	11.5 µm	16.0 µm	21.7 µm
AlN-BN	6.92 µm	9.19 µm	9.49 µm

**Table 5:** Wear coefficients calculated from the volume loss at 9.42 m/min.

Material	k × 10^−6^ (mm^3^ N^−1^ m^−1^)
Pure hBN	11.331
M26 BN	17.115
Macor	1.355
AlN-BN	0.455
3YSZ	0.02
Si_3_N_4_	0.021
Al_2_O_3_	0.018

## Data Availability

Data are contained within the article.
